# Concordance of apolipoprotein B concentration with the Friedewald, Martin-Hopkins, and Sampson formulas for calculating LDL cholesterol

**DOI:** 10.11613/BM.2022.010704

**Published:** 2021-12-15

**Authors:** Pieter-Jan Briers, Michel R. Langlois

**Affiliations:** 1 Department of Laboratory Medicine, AZ St-Jan Hospital, Brugge, Belgium; 2 Department of Diagnostic Sciences, Faculty of Medicine and Health Sciences, Ghent University, Ghent, Belgium

**Keywords:** apolipoprotein B, LDL-cholesterol, Friedewald formula, Martin-Hopkins formula, Sampson formula

## Abstract

**Introduction:**

Two new formulas, the Martin-Hopkins and the Sampson formula, were recently developed to overcome shortcomings of the Friedewald formula for calculating LDL-cholesterol. We aimed to compare the concordance of the two formulas with apolipoprotein B (apoB), a surrogate marker of the number of LDL particles.

**Materials and methods:**

In a study of serum lipid data of 1179 patients who consulted the AZ St-Jan Hospital Bruges for cardiovascular risk assessment, the correlation and concordance of the Friedewald, Martin-Hopkins and Sampson formulas with apoB concentration, measured by immunonephelometry, were determined and compared.

**Results:**

The Martin-Hopkins formula showed significantly higher correlation coefficient than the Friedewald formula with apoB in the entire dataset and in patients with low LDL-cholesterol < 1.8 mmol/L. Both Martin-Hopkins and Sampson formulas yielded > 70% concordance of LDL-cholesterol with regard to treatment group classification based on population-equivalent thresholds of apoB in hypertriglyceridemic patients (2-4.5 mmol/L), with the highest concordance (75.6%) obtained using Martin-Hopkins formula *vs*. 60.5% with Friedewald formula.

**Conclusion:**

The Martin-Hopkins (and, to a lesser extent, Sampson) formula is more closely associated with the number of LDL particles than Friedewald formula. This, in combination with literature evidence of lesser accuracy of the Friedewald formula, is an argument to switch from Friedewald to a modified, improved formula.

## Introduction

The reduction of low-density lipoprotein cholesterol (LDLC) is the primary target of lipid-lowering therapies in cardiovascular prevention as stated by international guidelines based on overwhelming evidence of epidemiological studies, clinical trials, and meta-analysis (*1*-*3*).

In most laboratories LDLC is calculated using the Friedewald formula (LDL-F) as follows: LDLC = total cholesterol (TC) – high-density lipoprotein cholesterol (HDLC) – very low-density lipoprotein cholesterol (VLDLC) wherein VLDLC is estimated as triglycerides (TG)/2.2 in mmol/L (*4*). Thus, it is assumed there is a constant ratio between VLDLC and TG in the VLDL particles. This is not the case *in vivo*, especially at high TG concentrations (> 2 mmol/L). At TG > 4.5 mmol/L the formula will even become unusable, and it is recommended to use a direct enzymatic LDLC assay (*5*). As the prevalence of type 2 diabetes mellitus and obesity has increased, hypertriglyceridemia at which LDL-F is less accurate is more common (approximately 25% of the general population has TG > 2 mmol/L) (*4*). In addition, LDL-F estimates LDLC less accurately at low concentrations < 1.8 mmol/L (*6*). This shortcoming was not a major problem when the formula was drafted in the 1970s, but has become more important as updated guidelines recommend that LDLC concentrations < 1.8 mmol/L and < 1.4 mmol/L should be pursued for high- and very high-risk patients, respectively (*1*, *4*). Furthermore, low LDLC concentrations have become more prevalent after the introduction of new lipid-lowering therapies such as more potent statins (with or without ezetimibe) and especially the PCSK9 inhibitors (*1*). In response to these imperfections of the Friedewald formula, several alternative formulas were developed to improve LDLC estimation (*7*–*11*). Two recent examples are the Martin-Hopkins (LDL-MH) and the Sampson (LDL-S) formulas.

The LDL-MH formula, developed by Dr. Martin at Johns Hopkins University (USA), is based on LDL-F, and replaces the fixed TG/VLDLC factor 2.2 with a variable factor to estimate VLDLC. This factor is obtained from a 180-cell table in which the factor depends on the non-HDLC value and the TG value of the patient. LDL-MH has better concordance with respect to direct LDLC quantification and the ultracentrifugation reference method (beta-quantification) than LDL-F, especially at low LDLC < 1.8 mmol/L (*6*, *12*).

The LDL-S formula was set up by constructing a bivariate quadratic equation that estimates VLDLC (*10*). Subsequently, on the basis of beta-quantification LDLC values, an equation was drawn up in which the obtained VLDLC term (displayed between parentheses) was used:



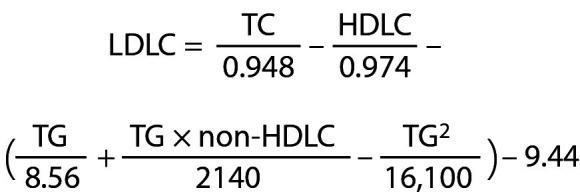



Sampson *et al.* reported that LDL-S estimates LDLC more accurately than LDL-F and LDL-MH in patients with hypertriglyceridemia. In addition, LDL-S is more accurate than LDL-F at low LDLC concentrations. In patients with normolipidemia, results were similar to those obtained with LDL-F and LDL-MH (*10*).

The novel formulas have been validated in studies by comparison with LDLC measured either directly or with the ultracentrifugation reference method (beta-quantification). While LDLC is a measurement of the cholesterol content in LDL particles, this does not reflect the number of atherogenic LDL particles in the circulation. In this study, we evaluated the relationship of the three aforementioned formulas with apolipoprotein B (apoB) concentration, used as a surrogate marker of the number of LDL particles (*4*). Because apoB and LDLC tests do not quantify the same measurand, the discordance of test results is inevitable, especially in people with predominant small cholesterol-depleted LDL particles such as in those with hypertriglyceridemia, diabetes, obesity, and metabolic syndrome (*13*). In these people, LDLC (estimated using LDL-F in most studies) does not always accurately predicts the risk of atherosclerotic cardiovascular disease (*13*, *14*). Guidelines recommend using apoB as an alternative treatment target and for risk assessment in these people (*1*). The aim of this study is to evaluate whether novel formulas to calculate LDLC may reduce discordance with apoB.

## Materials and methods

### Subjects

The study investigated lipid data of patients for which serum TC, TG, HDLC, LDLC, and apoB tests were requested in the AZ St.-Jan hospital Bruges, excluding patients with TG > 4.5 mmol/L. In total, data of 1179 fasting serum samples were included in the study and anonymized. The mean age of the population was 56 years (range: 37-72), 52% of the subjects were women. The samples were drawn in the phlebotomy unit of the hospital and represent a heterogeneous group of outpatients with and without dyslipidemia. The ethics committee of the AZ St.-Jan hospital Bruges approves research on residual material and anonymized data.

### Methods

On a Cobas 8000 analyser from Roche Diagnostics (Mannheim, Germany) TC, TG, and HDLC were quantified. Total cholesterol was quantified with an enzymatic colorimetric assay using cholesterol esterase and cholesterol oxidase. High-density lipoprotein cholesterol was determined with a direct enzymatic colorimetric assay in which non-HDL lipoproteins are combined with polyanions and detergents forming a water-soluble complex which is blocked from subsequent enzymatic reactions. The glycerol fraction of TG was quantified enzymatically after free glycerol blanking. ApoB was assayed by immunonephelometry on a BN-Prospec instrument from Siemens (Munich, Germany). LDL-F was automatically calculated and reported by the laboratory informatics system MOLIS version 4.41 from CompuGroup Medical (Koblenz, Germany). LDL-MH and LDL-S were calculated using Excel (Microsoft, Redmont, USA). Non-HDLC was calculated as TC – HDLC to enable LDL-MH and LDL-S calculations. Characteristics of the dataset are displayed in Table 1.

**Table 1:** Characteristics of the study dataset

	**Median**	**95% CI for the median**	**IQR**	**2.5–97.5 P**
TC (mmol/L)	5.3	5.2–5.3	4.6–6.0	3.1–7.4
TG (mmol/L)	1.1	1.0–1.1	0.8–1.7	0.5–3.4
HDLC (mmol/L)	1.5	1.5–1.6	1.2–1.9	0.7–2.6
Non-HDLC (mmol/L)	3.7	3.6–3.8	3.0–4.4	1.8–5.7
LDL-F (mmol/L)	3.1	3.0–3.1	2.4–3.8	1.3–5.1
LDL-S (mmol/L)	3.1	3.1–3.2	2.5–3.8	1.3–5.2
LDL-MH (mmol/L)	3.1	3.0–3.2	2.5–3.8	1.3–5.1
ApoB (g/L)	0.93	0.91–0.94	0.77–1.10	0.50–1.44
CI – confidence interval. IQR – interquartile range. P – population percentile. TC – total cholesterol. TG – triglycerides. HDLC – high-density lipoprotein cholesterol. LDL – low-density lipoprotein cholesterol. LDL-F – LDL calculated using the Friedewald formula. LDL-S – LDL calculated using Sampson formula. LDL-MH – LDL calculated using Martin-Hopkins formula. ApoB – apolipoprotein B.				

### Concordance/discordance analysis

For concordance analysis, we used treatment thresholds (therapeutic goals) to stratify the data. The thresholds recommended by the European Society of Cardiology and European Atherosclerosis Society (ESC/EAS) 2019 guidelines were used for LDLC: < 1.4 mmol/L (very high risk), < 1.8 mmol/L (high risk), < 2.6 mmol/L (moderate risk) and < 3.0 mmol/L (low risk) (*1*). This guideline also proposes treatment goals for apoB as an alternative target: < 0.65 g/L (very high risk), < 0.80 g/L (high risk) and < 1.00 g/L (moderate risk) (*1*). For people at low risk, no apoB treatment goal is recommended by the ESC/EAS guideline. Therefore, the threshold of 1.20 g/L for elevated apoB was used in this risk category (*15*).

To test the hypothesis that ESC/EAS-recommended apoB thresholds may not correspond to the population percentiles equivalent to LDLC, the concordance analysis was repeated with the same dataset using population-equivalent percentiles of apoB linked to the different risk groups. ApoB was matched to LDLC percentiles reported from the National Health and Nutrition Examination Survey (NHANES) population database (*16*, *17*). Low-density lipoprotein cholesterol thresholds of 1.4, 1.8, 2.6, and 3.0 mmol/L corresponded to the 3rd, 7th, 33rd, and 52nd percentile, respectively, in NHANES (*16*). The corresponding apoB concentrations in the same population percentiles were 0.50 g/L (3rd percentile), 0.60 g/L (7th percentile), 0.80 g/L (33rd percentile), and 0.90 g/L (52nd percentile) after rounding to the nearest 0.05 g/L (*16*). These values were used as population-equivalent apoB thresholds in the concordance analysis (Supplementary material, Table 1).

### Statistical analysis

The normality of the data was tested with the Shapiro-Wilk test. Spearman correlation coefficients were used to study the relationship between apoB and LDLC calculated with the three aforementioned formulas. The correlation coefficients were compared with each other with a z-test on Fisher z-transformed correlation coefficients. Correlations were investigated in the entire dataset, a subgroup with low LDL-F < 1.8 mmol/L, and a subgroup with TG 2.0–4.5 mmol/L. The LDL-F threshold of 1.8 mmol/L corresponds to the high-risk treatment target for LDLC and approximately the 2.5th percentile of general populations (*4*, *14*). The hypertriglyceridemia subgroup was defined according to the EAS-recommended TG threshold of 2 mmol/L (*18*). The number of concordant and discordant LDLC results relative to the apoB concentration was statistically compared using the chi-square test and Kappa statistics for agreement. To compare the different LDLC formulas with each other, Passing-Bablok (PB) regression was used. Median LDLC values were compared using the Kruskal-Wallis test. When a significant difference was detected, the test was combined with a *post-hoc* Conover analysis for pairwise comparison between the formulas. Two-tailed P-values less than 0.05 were considered statistically significant. The statistical analyses were performed using MedCalc software version 17.6 (Ostend, Belgium).

## Results

### Correlation between apoB and LDLC formulas

Normally distributed were LDL-F and LDL-S and LDL-MH was not. Spearman correlation coefficients between apoB and LDL-F, LDL-S and LDL-MH are shown in Table 2. The correlation coefficient between LDL-MH and apoB is significantly higher than the correlation coefficient between LDL-F and apoB (P = 0.001). The correlation coefficient of LDL-S with apoB is not significantly different from the correlation coefficient between LDL-F and apoB (P = 0.087).

**Table 2:** Spearman correlation coefficients between apoB concentration and the different LDLC formulas in the entire data set, the subgroup with LDLC < 1.8 mmol/L, and the subgroup with TG > 2 mmol/L

	**apoB**	
	**Correlation coefficient (95% CI)**	**P**
**Entire dataset (N = 1179)**		
LDL-F	0.85 (0.83–0.86)	< 0.001
LDL-S	0.86 (0.85–0.88)	< 0.001
LDL-MH	0.89 (0.87–0.90)	< 0.001
**LDL-F < 1.8 mmol/L (N = 115)**		
LDL-F	0.41 (0.24–0.55)	< 0.001
LDL-S	0.60 (0.47–0.71)	< 0.001
LDL-MH	0.67 (0.56–0.76)	< 0.001
**TG > 2 mmol/L (N = 238)**		
LDL-F	0.86 (0.82–0.89)	< 0.001
LDL-S	0.86 (0.82–0.89)	< 0.001
LDL-MH	0.87 (0.85–0.90)	< 0.001
CI – confidence interval. ApoB – apolipoprotein B. LDL – low-density lipoprotein cholesterol. LDL-F – LDL calculated using the Friedewald formula. LDL-S – LDL calculated using Sampson formula. LDL-MH – LDL calculated using Martin-Hopkins formula. TG – triglycerides.		

In the subgroup with LDL-F < 1.8 mmol/L, the correlation coefficients are notably lower than in the entire dataset. As in the entire dataset, LDL-MH has a significantly higher correlation coefficient with apoB than LDL-F has (P = 0.004). Again, there is no significant difference between LDL-S and LDL-F with regard to their correlation coefficient with apoB (P = 0.504).

In the subgroup with TG > 2 mmol/L, no significant differences in correlation coefficient with apoB were observed between the three formulas.

### Concordance analysis based on ESC/EAS thresholds

**Figure 1:**
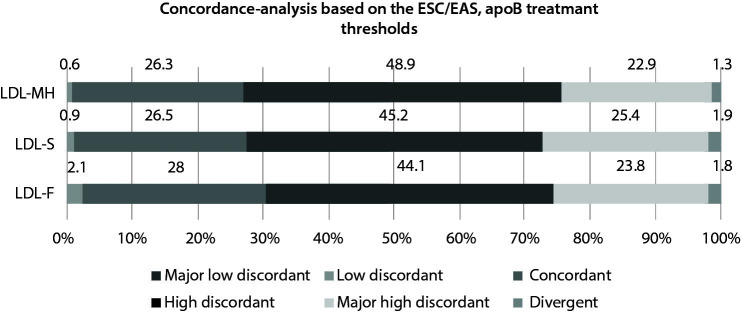
Percentages of LDLC results that were concordant, high discordant, low discordant, major high discordant, major low discordant and divergent from the apoB treatment thresholds in the concordance analysis based on ESC/EAS-recommended risk thresholds for apoB and LDLC. Discordant means that LDLC classifies the results 1 category too high or too low, and major discordant means that LDLC classifies the results more than 1 category too high or too low, compared to apoB. Divergent values are misclassified more than 2 risk categories. ESC/EAS – European Society of Cardiology and European Atherosclerosis Society. LDL – low-density lipoprotein cholesterol. LDL-MH – LDL calculated using Martin-Hopkins formula. LDL-S – LDL calculated using Sampson formula. LDL-F – LDL calculated using the Friedewald formula. ApoB – apolipoprotein B.

Concordance/discordance analysis based on ESC/EAS-recommended apoB thresholds showed low concordance between apoB and LDL-F, LDL-S or LDL-MH: < 30% (Figure 1). Strikingly, LDL-F, LDL-S, and LDL-MH systematically classify results in a higher risk category than the risk group based on apoB thresholds (Figure 1 and Supplementary material, Table 2). This leads to high percentages of high discordant LDLC results. For example, 67.9% of LDL-F results, among which 23.8% are classified more than 1 risk category too high, are discordantly higher than those based on apoB thresholds of the ESC/EAS guideline. No significant differences were observed in concordant LDLC results between the different formulas (Supplementary material, Table 3).

**Table 3:** Passing-Bablok regressions between the different LDLC formulas for the complete dataset and the subgroups with low LDLC and hypertriglyceridemia

	**PB regression**	**slope**	**intercept**	**R**	**P**
		**95% CI**			
**Entire dataset (N = 1179)**					
LDL-F *vs.* LDL-MH	LDL-F = 1.00 LDL-MH - 0.03	1.00 to 1.00	0.03 to 0.03	0.987	< 0.001
LDL-F *vs.* LDL-S	LDL-F = 0.98 LDL-S - 0	0.98 to 0.99	- 0.02 to 0.01	0.998	< 0.001
LDL-S *vs.* LDL-MH	LDL-S = 1.03 LDL-MH - 0.03	1.03 to 1.03	- 0.04 to - 0.02	0.995	< 0.001
**LDL-F < 1.8 mmol/L (N = 115)**					
LDL-F *vs.* LDL-MH	LDL-F = 0.95 LDL-MH + 0.05	0.88 to 1.00	- 0.03 to 0.17	0.768	< 0.001
LDL-F *vs.* LDL-S	LDL-F = 1.00 LDL-S - 0.03	0.92 to 1.00	- 0.03 to 0.10	0.906	< 0.001
LDL-S *vs.* LDL-MH	LDL-S = 1.00 LDL-MH + 0	0.96 to 1.00	0.00 to 0.06	0.946	< 0.001
**TG > 2.0 mmol/L (N = 238)**					
LDL-F *vs.* LDL-MH	LDL-F = 1.09 LDL-MH - 0.54	1.07 to 1.10	- 0.60 to - 0.49	0.983	< 0.001
LDL-F *vs.* LDL-S	LDL-F = 1.05 LDL-S - 0.34	1.05 to 1.06	- 0.37 to - 0.32	0.999	< 0.001
LDL-S *vs.* LDL-MH	LDL-S = 1.04 LDL-MH - 0.21	1.03 to 1.05	- 0.24 to - 0.18	0.992	< 0.001
CI – confidence interval. LDL – low-density lipoprotein cholesterol. LDL-F – LDL calculated using the Friedewald formula. LDL-S – LDL calculated using Sampson formula. LDL-MH – LDL calculated using Martin-Hopkins formula. TG – triglycerides. PB – Passing-Bablok.					

### Concordance analysis based on population-equivalent apoB percentiles

**Figure 2:**
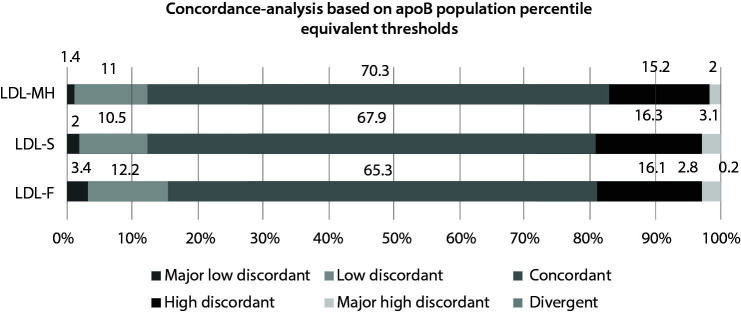
Percentages of LDLC results that were concordant, high discordant, low discordant, major high discordant, major low discordant and divergent from the population percentile-equivalent apoB thresholds. Discordant means that LDLC classifies the results 1 category too high or too low, and major discordant means that LDLC classifies the results more than 1 category too high or too low, compared to apoB. Divergent values are misclassified more than 2 categories. ApoB – apolipoprotein B. LDL – low-density lipoprotein cholesterol. LDL-MH – LDL calculated using Martin-Hopkins formula. LDL-S – LDL calculated using Sampson formula. LDL-F – LDL calculated using the Friedewald formula.

Concordance analysis using population-equivalent percentiles as apoB thresholds shows a concordance of 65-70% for LDL-F, LDL-S, and LDL-MH (Figure 2 and Supplementary material, Table 4). This analysis yielded less high discordant results than those based on ESC/EAS-recommended apoB thresholds (Figure 1). LDL-MH shows a significantly higher number of concordant results than LDL-F (P = 0.008). No significant differences in concordance were found between the LDL-F and LDL-S formulas and between the LDL-MH and LDL-S formulas (Supplementary material, Table 3). Kappa values (95% confidence interval) of agreement were 0.64 (0.61-0.67) for LDL-F, 0.67 (0.64-0.70) for LDLS, and 0.71 (0.68-0.74) for LDL-MH.

**Figure 3:**
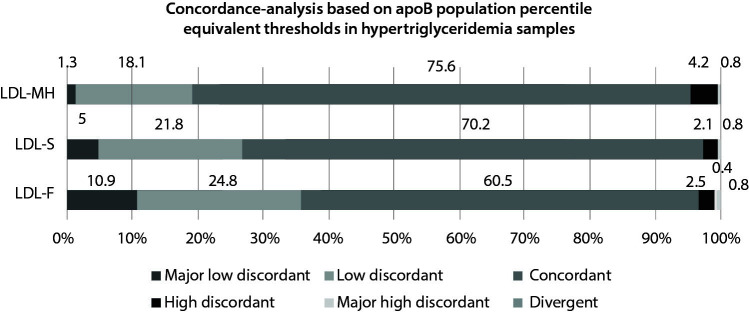
Percentages of LDLC results that were concordant, high discordant, low discordant, major high discordant, major low discordant and divergent from the population percentile-equivalent apoB thresholds in the concordance/discordance analysis of data in hypertriglyceridemic samples only (TG > 2 mmol/L). Discordant means that LDLC classifies the results 1 category too high or too low, and major discordant means that LDLC classifies the results more than 1 category too high or too low, compared to apoB. Divergent values are misclassified more than 2 categories. ApoB – apolipoprotein B. LDL – low-density lipoprotein cholesterol. LDL-MH – LDL calculated using Martin-Hopkins formula. LDL-S – LDL calculated using Sampson formula. LDL-F – LDL calculated using the Friedewald formula.

In the subgroup with TG > 2 mmol/L, apoB has > 70% concordance with LDL-MH or LDL-S and 60.5% with LDL-F (Figure 3 and Supplementary material, Table 5). The number of concordant results for LDL-MH was significantly higher than for LDL-F (P = 0.001). In contrast to the entire dataset, in the hypertriglyceridemic subgroup LDL-S also yields significantly more concordant results than LDL-F (P = 0.027) (Supplementary material, Table 3). Kappa values (95% confidence interval) of agreement were 0.46 (0.37-0.54) for LDL-F, 0.57 (0.49-0.66) for LDL-S, and 0.65 (0.56-0.73) for LDL-MH in this subgroup.

### Comparison between the LDLC formulas

Passing-Bablok regressions showed no significant differences between the different formulas in the entire dataset and in the subgroup with LDLC < 1.8 mmol/L (Table 3), with the exception of a small proportional difference of 1.7% between LDL-F and LDL-S and a small systematic difference between LDL-S and LDL-MH in the entire dataset. No significant differences in median LDLC concentrations were observed between the formulas at low LDLC (< 1.8 mmol/L) as well as full LDLC concentration range.

In the subgroup with TG > 2.0 mmol/L, the formulas are no longer interchangeable. Here, LDL-MH and LDL-S show a significant systematic difference of + 0.5 mmol/L and + 0.3 mmol/L, respectively, compared to LDL-F (Table 3). Furthermore, PB regression shows a significant proportional difference of 8.8% between LDL-F and LDL-MH, 5.3% between LDL-F and LDL-S, and 3.7% between LDL-MH and LDL-S. Finally, there is also a significant difference in median LDLC concentration between LDL-F (3.0 mmol/L) and LDL-MH (3.3 mmol/L) in hypertriglyceridemic samples (P = 0.012).

## Discussion

In this study of lipid data from outpatients attending the hospital for cardiovascular risk assessment, we found that the choice of the formula used to calculate LDLC may influence the concordance of LDLC- with apoB-based risk group classification. However, this concordance is largely dependent on whether guideline-recommended or population percentile-equivalent apoB thresholds are used.

Concordance/discordance analysis of treatment group classification based on thresholds of the ESC/EAS guidelines showed very low concordance of apoB (< 30%) with LDL-F, LDL-MH, and LDL-S. LDLC calculated with all formulas classified ≥ 50% of patients in a higher risk category than would be obtained based on apoB thresholds. This implies that, in more than half of the patients, lipid-lowering treatment would be initiated or intensified based on LDLC while deemed unnecessary based on apoB.

Concordances of the three LDLC formulas improved (65-70%) using population percentile-equivalent apoB thresholds and, as a consequence, the numbers of high discordant LDLC results were drastically reduced. This observation supports the proposal that using apoB thresholds based on population percentiles equivalent to LDLC would better match the LDLC treatment goals than the current ESC/EAS-recommended apoB thresholds (*19*). The current apoB thresholds are arbitrary and not well established as LDLC thresholds with regard to their clinical performance (risk stratification) in randomized trials and meta-analyses (*20*–*22*). They may need to be adjusted to reflect the same risk as in the eponymous LDLC-based treatment group (*19*). For example, the ESC/EAS-recommended high risk target of apoB (0.80 g/L) corresponds to approximately the 25th-30th population percentile while the risk group-equivalent LDLC threshold (1.8 mmol/L) lies between the 2nd and 8th percentile of populations (*4*, *14*, *16*). Indeed, it has been demonstrated that after meeting the high risk LDLC target, the clinical utility of apoB as a secondary target in addition to LDL-MH was limited when the ESC/EAS threshold value was used (*23*, *24*). The thresholds are ideally chosen in a prospective clinical performance study evaluating which values most accurately classify patients within the appropriate risk category (*25*). The population percentile-equivalent thresholds used in this study are from a U.S. general population survey (*16*) and were used for illustrative purpose only.

In hypertriglyceridemic samples, the percentage concordant results for LDL-F (60.5%) compared to population percentile-equivalent apoB thresholds were significantly lower than with the other two formulas (> 70%). In addition, in the hypertriglyceridemic subgroup, the three formulas yield lower discordant LDLC results and less high discordant LDLC results than in the entire dataset. This is most pronounced for LDL-F which underestimates the risk of 36% of patients compared to apoB-based classification, while LDL-MH underestimates < 20% of risk categories. This observation confirms that the Friedewald formula estimates LDLC less accurately than LDL-S and LDL-MH compared to ultracentrifugation-LDLC (beta-quantification) at high TG concentrations (*10*, *12*). In this case, the VLDL particles will contain relatively more TG and the fixed TG/VLDLC ratio of 2.2 used in LDL-F will not sufficiently correct for the increased TG concentration, leading to underestimation of LDLC (*13*).

Especially in the subgroup with LDLC < 1.8 mmol/L, the correlation coefficient between LDL-F and apoB is lowest. This may be explained by a lesser accuracy of LDL-F using the fixed VLDLC term at a low LDLC concentration range, in which VLDLC represents a relatively larger fraction of the total serum cholesterol (*6*, *10*, *12*, *24*). In contrast, LDL-MH using the adjustable VLDLC term shows a significantly higher correlation coefficient with apoB in the subgroup with LDLC < 1.8 mmol/L and in the entire dataset, compared to LDL-F.

Like the concordance-analysis, PB regression analysis between the formulas suggests that LDL-F underestimates LDLC in patients with hypertriglyceridemia. In the subgroup with low LDLC < 1.8 mmol/L, no significant systematic differences were observed between the three formulas which are in contradiction to what has been described in the literature by comparison with ultracentrifugation-LDLC (*10*, *12*, *24*). Compared with the beta-quantification reference method, the LDL-S formula was more accurate than LDL-F and LDL-MH formulas for patients with hypertriglyceridemia, whereas both LDL-S and LDL-MH performed better than LDL-F at low LDLC concentrations (*10*). However, in our study only a pairwise comparison between the formulas was performed and no comparison was made with the LDLC reference method. Therefore, from our data, no statements can be made about the accuracy of the formulas to calculate LDLC.

Our results suggest that a more accurate LDLC formula leads to a better concordance with the number of atherogenic lipoprotein particles – assessed by apoB assay. ApoB has been proposed as a better predictor of cardiovascular risk than LDLC in patients with mild-to-moderate hypertriglyceridemia, diabetes, and metabolic syndrome characterized by high numbers of small dense LDL particles (*13*). These small dense LDL particles are cholesterol-depleted and, therefore, the associated risk is better captured by apoB, which is a measure of LDL particle number, rather than LDLC, which is a measure of cholesterol concentration in the particles (*26*, *27*). Besides the fact that LDL-MH and LDL-S have been recommended as more accurate formulas than LDL-F (*4*, *24*), this is an additional reason to consider switching to a novel, improved formula as a predictor of the atherosclerotic cardiovascular risk attributable to LDL (*11*, *24*), when apoB is not available.

Because apoB and LDLC do not reflect the same measurand, discordance is inevitable regardless of the formula used to calculate LDLC, despite the higher correlation coefficient obtained between LDL-MH and apoB in this study. In addition to apoB from LDL particles, fasting apoB measurement also includes the apoB from VLDL particles and Lp(a) (*13*, *14*). As a result, apoB concentration will always be a slight overestimation of the number of LDL particles (*14*), although the number of VLDL particles in most patients with hypertriglyceridemia will not exceed 10% of the total number of LDL particles (*13*). Furthermore, patients with low LDLC are on lipid-lowering therapy which reduces the cholesterol concentration in LDL particles, but to a lesser extent decreases the number of LDL particles (*28*), and statins do not reduce Lp(a).

### Limitations of the study

The anonymized dataset used for this study does not include clinical information of the subjects such as medication, apart from gender and age. Information on lipid-lowering treatment would be useful because it affects the lipid profile of individuals. However, no statements can be made about such an effect which limits the generalizability of the study.

The study excluded TG values 4.5–9.0 mmol/L because in this concentration range the Friedewald and Martin-Hopkins formulas, unlike the Sampson formula, are unusable and it is recommended to use a direct enzymatic LDLC assay (*5*). It would be valuable to evaluate the relationship between apoB and LDL-S in this concentration range (*10*).

The formulas used data from TC, TG, and HDLC measurements with Roche reagents. It would have been interesting to use different analytical platforms because it has been demonstrated that the analytical platform and reagent used have an effect on the performance of the formulas, especially for LDL-S with Roche reagents (*29*, *30*).

## Conclusion

Novel LDL-MH and, to a lesser extent, LDL-S formula improve the concordance of calculated LDLC with LDL particle numbers estimated by apoB. This, in combination with literature evidence of lesser accuracy of LDL-F, is an additional argument to switch from classical Friedewald to a modified, improved formula as common laboratory practice for reporting LDLC. Switching to LDL-MH or LDL-S can be achieved without introducing an additional cost because the same variables from the standard lipid profile are used in the novel formulas as in the Friedewald formula.
